# A Val85Met Mutation in Melanocortin-1 Receptor Is Associated with Reductions in Eumelanic Pigmentation and Cell Surface Expression in Domestic Rock Pigeons (*Columba livia*)

**DOI:** 10.1371/journal.pone.0074475

**Published:** 2013-08-15

**Authors:** Michael W. Guernsey, Lars Ritscher, Matthew A. Miller, Daniel A. Smith, Torsten Schöneberg, Michael D. Shapiro

**Affiliations:** 1 Department of Biology, University of Utah, Salt Lake City, Utah, United States of America; 2 Institute of Biochemistry, Faculty of Medicine, University of Leipzig, Leipzig, Germany; 3 Department of Chemistry, Goshen College, Goshen, Indiana, United States of America; University of Florence, Italy

## Abstract

Variation in the *melanocortin-1 receptor* (*Mc1r*) is associated with pigmentation diversity in wild and domesticated populations of vertebrates, including several species of birds. Among domestic bird species, pigmentation variation in the rock pigeon ( *Columba* *livia* ) is particularly diverse. To determine the potential contribution of *Mc1r* variants to pigment diversity in pigeons, we sequenced *Mc1r* in a wide range of pigeon breeds and identified several single nucleotide polymorphisms, including a variant that codes for an amino acid substitution (Val85Met). In contrast to the association between Val85Met and eumelanism in other avian species, this change was associated with pheomelanism in pigeons. *In vitro* cAMP accumulation and protein expression assays revealed that Val85Met leads to decreased receptor function and reduced cell surface expression of the mutant protein. The reduced *in vitro* function is consistent with the observed association with reduced eumelanic pigmentation. Comparative genetic and cellular studies provide important insights about the range of mechanisms underlying diversity among vertebrates, including different phenotypic associations with similar mutations in different species.

## Introduction

Variation in the *melanocortin-1 receptor* (*Mc1r*) is associated with pigment variation across a phylogenetically broad range of wild and domestic populations of vertebrates, including mammals, fishes, reptiles, and birds (e.g., [1–4]). *Mc1r* encodes a 7-span transmembrane G protein-coupled receptor, and in melanocytes it plays a major role in regulating the production of eumelanin (brown and black pigments). In general, stimulation of the receptor by its endogenous agonist α-melanocyte stimulating hormone (αMSH) results in increased production of eumelanin, while binding with the inverse agonist Agouti protein results in decreased eumelanism and/or increased production of pheomelanin (red and yellow pigments). Depending on the domain of the protein affected by mutations, changes to Mc1r localization and function can lead to eumelanic (increased activity), pheomelanic, or blanched phenotypes (decreased activity) [3], and all confirmed cases of *Mc1r*-mediated pigment variation affect the coding sequence of the gene.

Among vertebrates, birds display a spectacular range of feather pigmentation phenotypes. Variation in color type (as opposed to pattern) largely reflects variation in the production of eumelanic versus pheomelanic pigments by feather melanocytes. Birds exhibit widespread color variation among species, and less commonly, they show substantial variation *within* species. In the latter cases, amino acid substitutions in Mc1r are associated with color polymorphisms in several distantly related species, including both wild [5–11] and domestic populations [12–17]. In the wild, *Mc1r*-mediated pigment variation is associated with differences in mate choice (e.g., [6,9]), thermoregulation [5], and immune system response [10]. Strikingly, most derived alleles of *Mc1r* described to date in birds are associated with increased deposition of eumelanin (but see 12), even in cases of deletions and truncated proteins, which tend to result in pheomelanic pelage in mammals [2,10,11,16].

**Figure 1:**
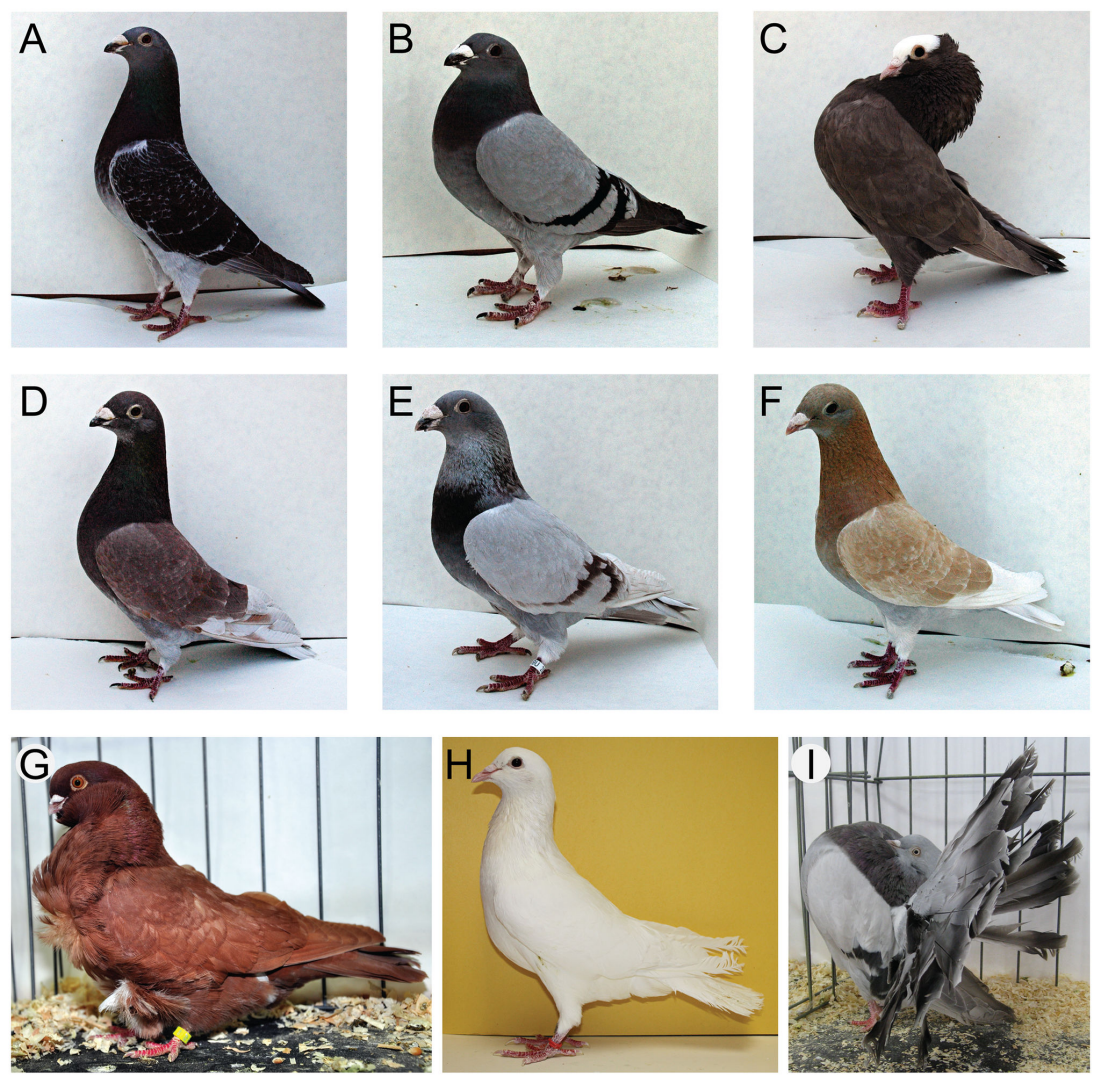
Examples of plumage pigment variation in domestic rock pigeons. All birds are show homer breed unless otherwise noted. **A**–**C**. Eumelanic phenotypes: **A**, black check; **B**, blue bar (same color as **A**, but different pattern); **C**, brown (Mookee). **D**–**G**. Pheomelanic phenotypes: **D**, ash-red check; **E**, ash-red bar (same color as **D**, but different pattern); **F**, yellow check (dilute form of phenotype in **D**); **G**, *recessive red* (Chinese owl). **H**. White (white carneau). **I**. Fantail, a breed examined in subset of association tests. Panels **H** and **I** modified after [27,48], respectively; photos courtesy of Eric Domyan (**A**–**F**) and Sydney Stringham (**H**).

Pigmentation variants are among the first traits to incur strong selection in domestic birds [18], and within-species variation in the rock pigeon ( *Columba* *livia* ) is particularly dramatic (Figure 1). Pigeons vary in pigmentation type and pattern in free-living [19–21] and domestic populations, where color is under intensive artificial selection in exhibition breeds [22,23]. The Mendelian inheritance patterns of several color traits has been thoroughly studied by scientists and hobbyists alike, and the three principal colors – blue (the ancestral gray to black color typically seen in feral pigeons), ash-red, and brown – are known to be allelic at a single sex-linked locus [23]. A recessive allelic variant at an autosomal locus can also confer pheomelanic plumage, and this *recessive red* locus is epistatic to the major sex-linked color locus. Several other autosomal and sex-linked loci contribute to the intensity and pattern of plumage pigment deposition. However, despite longstanding interest in pigmentation genetics in domestic pigeons [23–26] the molecular origins of color variation remain poorly understood.

Here, we report sequence variation in *Mc1r* among domestic pigeons and test for associations between genotypes and melanic phenotypes. We also test the functionality of a mutant allele of *Mc1r* in pigeons, and discuss our findings in light of studies of this gene in other vertebrates.

## Methods

### Ethics statement

This study was approved by the University of Utah Institutional Animal Care and Use Committee, protocols 09-04015 and 10-05007. Private owners of birds signed a consent form allowing us to use blood and feathers in this study. Importation of feathers from outside the USA was approved under USDA APHIS permit 110106, issued to M.D. Shapiro. No material was collected from the wild for this study.

### Discovery of variant alleles

We collected blood and feather samples and extracted DNA as previously described [27]. We selected a panel of 120 birds with diverse pigment phenotypes (recorded in photographs by us or as reported by breeders who donated feather samples via mail) from 68 breeds to characterize polymorphisms in the coding sequence of *Mc1r*. We focused on well-described phenotypes [23] that could be easily classified as blue (dark grey/black, the ancestral color morph), ash-red, brown, or modified versions of these colors such as their dilute forms (e.g., silver/dun, ash-yellow, and khaki, respectively). We also included *recessive red* and white birds.

Most of the *Mc1r* coding sequence was amplified using the avian degenerate primers MSHR72 (5’ ATGCCAGTGAGGGCAACCA-3’) and MSHR9 (5’-CTGGCT CCGGAAGGCATAGAT-3’) [6]; however, this sequence excluded the 5’ and 3’ ends of the coding sequence. We amplified the 5’ region of *Mc1r* containing the start codon using the primers mc1r_F2 (5’-CTTTAAAGCGGGACAGAGAAA-3’) and mc1r_R4 (5’-GAAGAGGAAGAAGCTGATGAG-3’), followed by nested PCR using the same forward primer (mc1r_F2) and a different reverse primer nested just 5’ to mc1r_R4 (mc1r_R3 5’-GATGGCATTGTTGCGATAGTA-3’). The 3’ region of *Mc1r* containing the stop codon was amplified using the primers mc1r_F6 (5’-CACCTGCAGCTCTGTTGTGT-3’) and mc1r_R6 (5’-ATGCCATTATCGGTGTCCCAC-3’). PCR products were gel extracted (Qiaex II Gel Extraction kit, Qiagen, Valencia, CA) and Sanger sequenced by the University of Washington High Throughput Genomics Unit using the primers MSHR9, MSHR72, mc1r_F2, mc1r_R4, mc1r_F6, and mc1r_R6. Sequences were assembled and analyzed using Sequencher software (Genecodes, Ann Arbor, MI). Of the 120 original birds in the panel, 113 from 67 breeds yielded usable sequences (94.2% success rate).

### TaqMan Genotyping Assay

We genotyped a G253A mutation (coding for a Val85Met polymorphism) on 337 pigeons, including 77 birds used in the Sanger-sequenced SNP discovery sample, using a TaqMan assay with the following oligonucleotides: forward primer, 5’- CATCTGCTGCCTAGCCATCT C-3’; reverse primer, 5’-GCTCCATCAGCAGCATGAAGA-3’; probes 5’ VIC-ATGCTGGTGAGCGTC-3’ (Val allele) and 5’ FAM-CATGCTGATGAGCGTC-3’ (Met allele.) TaqMan assays were performed by the University of Utah DNA Genomics Core Facility using the manufacturer’s protocol (Applied Biosystems, Foster City, CA). We discarded samples with genotype calls that fell outside the 95 percent confidence interval, leaving a sample of 313 birds (92.9% success rate).

### Association Tests

To reduce the possibility of ambiguous phenotypes based solely on descriptive information from breeders, we further filtered the dataset to exclude birds for which we did not also have photographs. This reduced the sample to 237 birds. We performed a final filter on the data to avoid pseudo-replicates in the dataset: we included only one bird per breeder of each breed with the same color and genotype, leaving a total of 190 birds from 89 breeds. Pigeons were scored for pigmentation phenotypes based on two categorical schemes. First, birds were classified as either eumelanic or pheomelanic based on their plumage color. A bird was classified as eumelanic if its major plumage coloration was a shade of brown or black, including gray. Pheomelanic birds were those with red or yellow plumage. Second, birds were categorized as blue/black, ash-red, brown (color phenotypes at the major sex-linked pigmentation locus); *recessive red* (autosomal, recessive alleles, and epistatic to the major color locus); and white (most common locus is autosomal with recessive alleles and epistatic to the major locus, but other autosomal loci can contribute as well) [23]. This more specific classification allowed us to test for enrichment of mutant alleles in blue/black, *recessive red*, and white birds.

We used GraphPad Prism software (GraphPad Software, San Diego, CA) to conduct tests for association between phenotypes and *Mc1r* genotypes using a chi-square test with 2 X 3 contingency tables. White birds were excluded from analyses except for tests of white versus all other colors. We excluded white birds because all-white pigmentation can be a complicated phenotype requiring the interaction of multiple loci [23].

### Cloning of Val85 and Met85 alleles

We isolated and cloned Val85 and Met85 alleles of Mc1r to test the functional effects of the Val85Met substitution. We amplified alleles from genomic DNA of a black mottle West-of-England tumbler (Val85) and a white standard fantail (Met85) by PCR using primers containing *Hind*III and *Eco*RI restriction sites: Mc1r_F7_*Hind*III 5'-TCGATAAGCTTGTGCCCTGGAGCTGAGGT-3' (*Hind*III site in italics) and Mc1r_R7_*Eco*RI 5'-GTCACGAATTCGTGTCCCAC TGCCTACCAG-3' (*Eco*RI site in italics). Products of each of these reactions were then gel-purified (Qiaex II Gel Extraction kit, Qiagen) and cloned into the pcDNA3.1/V5-HisB vector (Invitrogen, Grand Island, NY). Allele sequences were confirmed to be identical at the nucleotide level except for the G253A SNP that codes for the variable Val85Met residue.

### Functional testing of alternative alleles

COS-7 and HEK293T cells were grown in Dulbecco’s modified Eagle’s medium (DMEM) and F12 medium, respectively, supplemented with 10% (v/v) fetal bovine serum, 100 units/ml penicillin and 100 µg/ml streptomycin, at 37°C in a humidified 5% CO_2_ incubator. For expression in mammalian cell lines, the two *Mc1r* alleles were subcloned into the mammalian expression vector pcDps. To quantify protein expression and the amount of plasma membrane-integrated Mc1r, the two *Mc1r* alleles were double-tagged with an N-terminal HA (haemagglutinin) tag and a C-terminal FLAG-tag. All PCR-derived constructs were verified by sequencing. Transient transfection experiments were performed with Lipofectamine 2000 (Invitrogen).

For cAMP assays, transfected HEK293T cells were split into a 384-well plate (10,000 cells per well) and stimulated with αMSH. Concentrations ranging from 0.1 nM to 100 µM were administered in logarithmic increments. The cAMP content of each well was determined by a non-radioactive cAMP accumulation assay based on the ALPHAScreen technology according to the manufacturer’s protocol (PerkinElmer LAS, Rodgau-Jügesheim, Germany). A third construct containing GFP, which should exhibit no cAMP accumulation, was used as a negative control for the assay. Cell vitality was measured using a 10-µM forskolin treatment to ensure that the adenylyl cyclase of all cells was capable of producing cAMP. Cyclic AMP accumulation data were analyzed using GraphPad Prism.

To assess the expression of full-length HA/FLAG double-tagged Mc1r proteins, and to demonstrate that the levels of cell surface expression were not due to a decrease or increase in receptor expression in general, a sandwich ELISA was used and performed as previously described to measure total cellular expression [28]. Specific optical density (OD) readings (OD_492/620 nm_ value of HA/FLAG-tagged construct minus OD_492/620 nm_ value of GFP-transfected cells) are given as a percentage of the Val85 allele of Mc1r. Results are reported as means ± SD of three independent experiments, each performed in triplicate or quadruplicate.

## Results

### Variation in Mc1r sequence

We identified 7 variants in the coding sequence of *Mc1r* in rock pigeons (Genbank accession numbers KF234242-KF234252). Of these coding variants, 3 were predicted to be synonymous and 4 non-synonymous at the amino acid level (Table 1). The most common haplotype was homozygous in 45.3% of birds, and heterozygous in 25.5%. When we further included haplotypes with only synonymous nucleotide differences, a total of 55.6% of birds were homozygous for the most common amino acid sequence, and another 36.8% were heterozygous. Four other haplotypes harbored a G253A nucleotide substitution, resulting in a Val85Met amino acid change (3.8% homozygous for A253, 7.5% heterozygous). When we examined variation within breeds, we noted that Met85 alleles were enriched in standard and Indian fantails (10% homozygous, 15% heterozygous), which are closely related to each other [27,29]. Both Val and Met are nonpolar and hydrophobic amino acids, but Met has a sulfur-containing side chain. The Val85 residue is highly conserved across vertebrates (Table 2), suggesting that it is probably important for normal Mc1r function [30].

**Table 1:** *Mc1r* coding sequence haplotypes in the domestic rock pigeon; amino acid numbering is identical to chicken.

Nucleotide	G253A	G279A	G343A	T354C	A520G	C649T	T840C		
Amino acid	V85M	Syn	D115N	Syn	S174G	R217C	Syn		
Haplotype								Hom^*^	Het^*^
1	G	G	G	T	A	C	T	45.3% (48)	25.5%(27)
2	.	.	.	.	.	.	C	10.4% (11)	11.3% (12)
3	.	A	.	.	G	.	.	8.5% (9)	0.9% (1)
4	A	.	.	.	.	.	.	2.8% (3)	5.7% (6)
5	.	.	.	.	.	T	.	2.8% (3)	4.7% (5)
6	.	.	A	.	.	.	.	0.9% (1)	2.8% (3)
7^†^	A	.	A	.	.	.	-	0.9% (1)	0% (0)
8	.	.	.	C	.	.	.	0.9% (1)	0% (0)
9	.	.	.	.	G	.	.	0% (0)	1.9% (2)
10	A	.	.	.	.	.	C	0% (0)	0.9% (1)
11	A	A	.	.	.	.	.	0% (0)	0.9% (1)
A^§^ 1	.	G/A	.	.	A/G	C/T	.	(1)	
A 2	.	G/A	.	.	A/G	.	.	(2)	
A 3	G/A	.	.	.	.	.	T/C	(1)	
A 4	.	.	.	.	.	.	-	(2)	
A 5	G/A	.	.	.	.	.	-	(1)	

* Hom, homozygous; Het, heterozygous; calculated as percentage of 106 birds with unambiguous haplotypes, numbers of birds in parentheses.

† Sequence is missing at synonymous T840C, but other SNPs show this is a distinct haplotype.

§ A, ambiguous: samples either have missing data at synonymous sites or haplotypes could not be reliably phased due to more than one SNP in the sequence; number of individuals in each category indicated in parentheses at right.

**Table 2:** Mc1r alignment around amino acid position 85 (relative to pigeon sequence) for 8 birds, 3 mammals, a lizard, and a fish.

Species*	**81**	**82**	**83**	**84**	**85**	**86**	**87**	**88**	**89**
*Columba* *livia* (Val85)	S	D	M	L	**V**	S	V	S	N
*Columba* *livia* (Met85)	.	.	.	.	**M**	.	.	.	.
*Anser* *caerulescens* (white)	.	.	.	.	**.**	.	.	.	.
*Anser* *caerulescens* (black)	.	.	.	.	**M**	.	.	.	.
*Gallus gallus*	.	.	.	.	**.**	.	.	.	.
*Anas platyrhynchos*	.	.	.	.	**.**	.	.	.	.
*Sula* *sula* (white)	.	.	.	.	**.**	.	I	.	.
*Sula* *sula* (brown)	.	.	.	.	**M**	.	I	.	.
*Crax* *alector*	.	.	.	.	**.**	.	I	.	.
*Coereba* *flaveola*	.	.	.	.	**.**	.	I	.	.
*Phylloscopus* *trochilus*	.	.	.	.	**.**	.	I	G	.
*Mus musculus*	.	.	L	M	**.**	.	.	.	I
*Canis familiaris*	.	.	L	.	**.**	.	.	T	.
*Camelus* *bactrianus*	.	.	L	.	**.**	.	M	.	.
*Sceloporusundulates*	.	.	.	.	**.**	.	I	.	.
*Danio rerio*	A	.	.	.	**.**	.	.	.	.

* Species key: *Columba* *livia* , rock pigeon; *Anser* *caerulescens* , snow goose; *Sula* *sula* , red-footed booby; *Crax* *alector* , black currasow; *Coereba* *flaveola* , bananaquit; *Phylloscopus* *trochilus* , willow warbler; *Gallus gallus*, red jungle fowl; *Mus musculus*, house mouse; *Canis familiaris*, domestic dog; *Camelus* *bactrianus* , Bactrian camel; *Sceloporus* *undulatus* , eastern fence lizard; *Danio rerio*, zebrafish.

### Association of Val85Met with pigment variation

**Figure 2:**
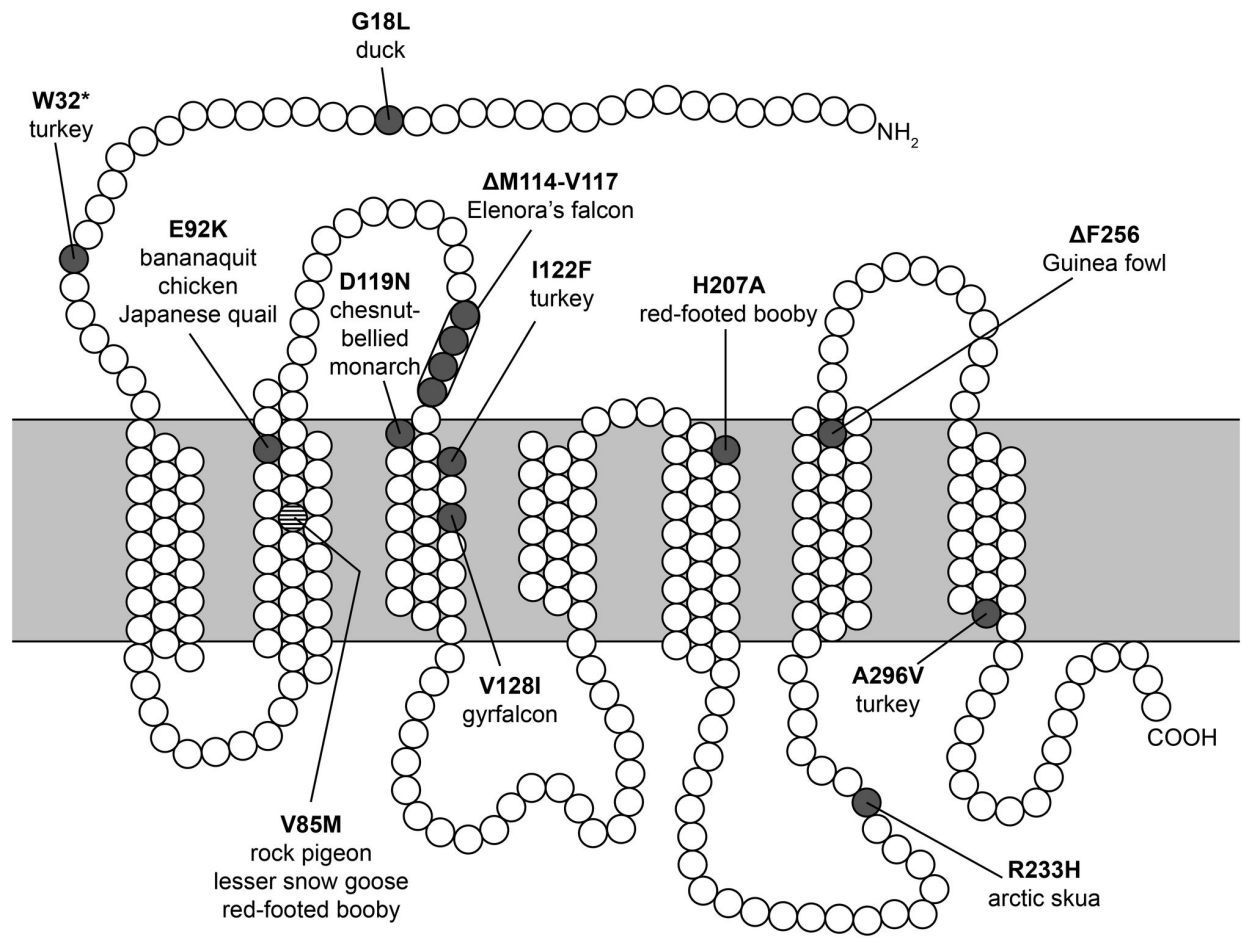
Amino acid sequence variation among avian orthologs of Mc1r. Several variants have been implicated in pigment variation within avian species. The Val85Met mutation found in domestic pigeons is associated with eumelanism in the lesser snow goose and red-footed booby. Functional studies of Mc1r protein variants have been conducted for chicken [14] and pigeon (this study). Additional mutations in chicken have been identified on the E92K background [13] but are not shown here. Figure based on previous review of avian Mc1r diversity [2].

**Figure 3:**
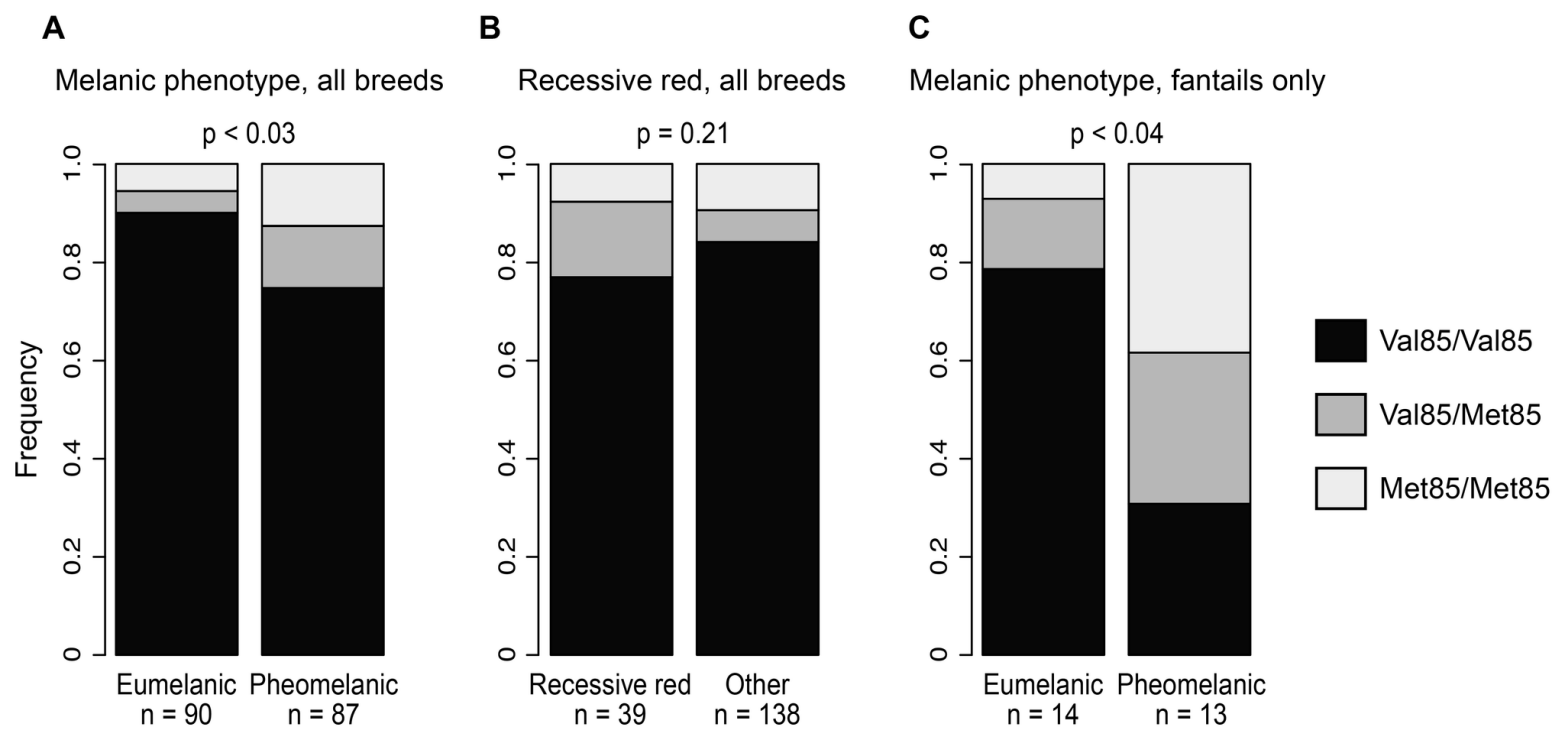
Met85 alleles of Mc1r are associated with pheomelanism in the domestic rock pigeon. **A**. Genotype frequencies across all sampled breeds, with birds categorized as eumelanic or pheomelanic. Met85 genotypes are significantly enriched in pheomelanic birds (p < 0.03, Chi-square test). **B**. Met85 genotypes are not significantly enriched in pheomelanic *recessive red* birds (p = 0.21, Chi-square test). **C**. Met85 genotypes are significantly enriched in pheomelanic standard and Indian fantails (p < 0.04, Chi-square test).

The Val85Met mutation in *Mc1r* is a particularly interesting candidate to influence pigment type in pigeons because it is strongly associated with eumelanic phenotypes in the lesser snow goose ( *Anser* *caerulescens* *caerulescens*) and red-footed booby ( *Sula* *sula* ), either alone or in combination with another amino acid substitution on the same haplotype, respectively [6,7] (Figure 2). Therefore, we predicted that this mutation would be enriched in pigeons with eumelanic plumage pigmentation, and depleted in pigeons with pheomelanic pigmentation. To examine this hypothesis, we genotyped 190 pigeons from 89 breeds at codon 85 to test for associations with melanic phenotypes. First, we tested for an association between *Mc1r* genotypes and completely white plumage, but did not find one (χ^2^ = 3.24, p = 0.20). Next, contrary to expectation, we did not find an enrichment of Met85 alleles in black or other eumelanic birds (Figure 3). However, we did find a significant enrichment of Met85 alleles in pheomelanic birds (eumelanic versus pheomelanic: χ^2^ = 7.22, p < 0.03, n = 177, white birds excluded; black/blue – the phenotypes containing the highest proportion of eumelanin [31] – versus brown and pheomelanic colors: χ^2^ = 3.27, p = 0.20; n = 177). We did not find an association between *Mc1r* genotypes and the pheomelanic *recessive red* phenotype (χ^2^ = 3.11, p = 0.21, n = 177). We can therefore infer that Val85Met in *Mc1r* does not underlie the *recessive red* phenotype.

We repeated the same association tests on a subset of our sample, the fantail breeds, in which Met85 alleles are found at a higher frequency than the overall sample. Here, too, we found an association between genotype at codon 85 and qualitative eumelanic versus pheomelanic phenotypic categories (χ^2^ = 6.57, p < 0.04, n = 27). As with the overall sample, we did not find an association between genotypes and all-white plumage (χ^2^ = 2.54, p = 0.28, n= 34; we did not have a sufficiently large sample of *recessive red* fantails to test for associations). When we removed the fantail sample from the species-wide sample, the association between *Mc1r* genotype and pheomelanic phenotype was not significant (χ^2^ = 3.77, p = 0.15, n = 150).

In summary, we found an association between Met85 alleles and pheomelanic phenotypes in domestic rock pigeons, both across 89 breeds and in fantail breeds; however, the association in the species-wide sample depends upon inclusion of the fantails. These findings contrast with previous studies in other bird species, in which Val85Met was strongly associated with eumelanic phenotypes [6,7].

### The Val85Met mutation reduces amount of Mc1r at the plasma membrane

**Figure 4:**
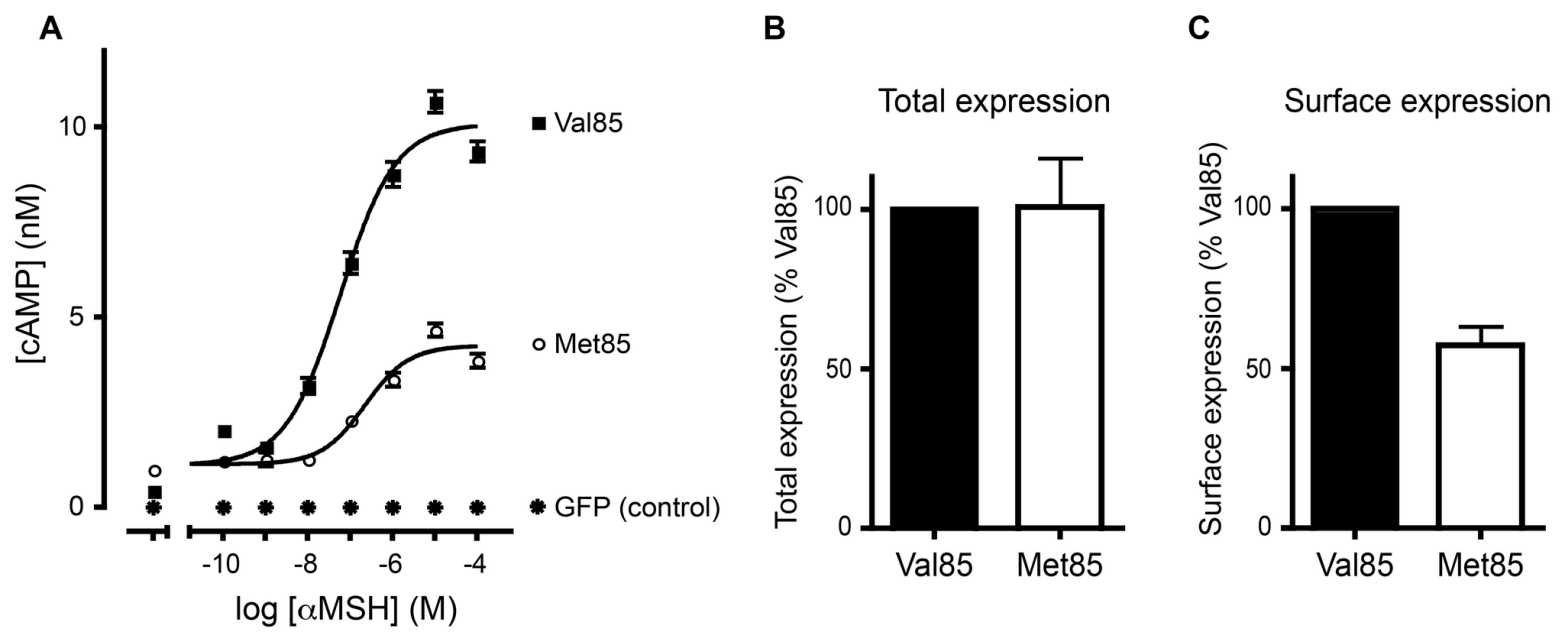
Functional differences between Val85 and Met85 alleles of Mc1r. **A**. cAMP production in response to αMSH stimulation is reduced in cells transfected with the Met85 allele relative to the Val85 allele (p < 0.02, paired t-test). Error bars, ± SEM. **B**. Total cellular protein expression of HA/FLAG-tagged Mc1r is equivalent in COS-7 cells transfected with pigeon Val85 and Met85 alleles. The non-specific OD_492/620 nm_ value (GFP) was 0.008 ± 0.001 (0% set point) and the OD_492/620 nm_ value of the Val85 allele was 0.415 ± 0.070 (100% set point). **C**. Surface protein expression of the Met85 allele is reduced relative to Val85 allele (p < 0.007, one-sample t-test). Non-specific OD_492/620 nm_ value (GFP) was 0.053 ± 0.050 (0%) and the OD_492/620 nm_ value of the Val85 allele was 0.501 ± 0.049 (100%).

Given the unexpected association between pheomelanism and Val85Met, we next tested the functionality of Val85 and Met85 alleles. Based on genetic associations with eumelanism in other birds, we would predict that the Met85 allele would have a higher functionality; however, based on the association with pheomelanism we found in pigeons, we would predict the opposite effect. To distinguish between these two possibilities, we transfected HEK293T cells with expression constructs coding for the most common *Mc1r* allele found in our variant discovery screen (Table 1), or an allele that differed only in the G253A SNP coding for the Val85Met polymorphism (no other synonymous or non-synonymous coding changes). We then assayed for production of cAMP in response to stimulation with the Mc1r agonist αMSH, and found that the Met85 allele produced significantly less cAMP relative to the Val85 allele (p < 0.02, paired t-test, n = 3 replicates; responses at highest agonist concentration: Val85 allele = 9.37 ± 1.41 pmol/mg protein, Met85 allele = 3.87 ± 0.50 pmol/mg protein) (Figure 4A). Because a decrease in cAMP production is correlated with pheomelanin synthesis [32], our functional tests suggest that the Met85 allele should not be enriched in eumelanic pigeons; indeed, this is consistent with our genetic association results.

To further elucidate the mechanism underlying the functional discrepancy between alleles, we examined the cellular localization of Mc1r proteins containing either Val85 or Met85. To do this, we transfected COS-7 cells with HA/FLAG-tagged versions of the two alternative *Mc1r* alleles, and quantified expression by sandwich ELISA. We found that both alleles were expressed at similar levels overall (Met85 allele expression = 100.7% of Val85 allele, SD = 15.3; Figure 4B), but cell surface expression of the Met85 allele was only 57.3% of the Val85 allele (p < 0.007, SD = 5.9, μ_O_ = 100, one-sample t-test) (Figure 4C). Together, these experiments suggest that the Val85Met mutation results in reduced Mc1r-mediated production of cAMP, and this reduced functionality is probably due, at least in part, to reduced localization of Mc1r proteins to the plasma membrane.

## Discussion

Our study of *Mc1r* and pigment diversity in domestic pigeons revealed a significant association between a Val85Met substitution and pheomelanism. This mutation was found on 4 haplotypes from 8 breeds in our SNP discovery set, and in 17 different breeds including our larger genotyping sample. Consistent with this genetic association, our functional studies showed that cells transfected with a Met85 allele produced significantly less cAMP when stimulated by the Mc1r agonist αMSH. However, genetic association tests confirm that Val85Met is not associated with the classic *recessive red* pheomelanic phenotype in pigeons, a known simple Mendelian trait [23]. The pigeon Met85 variant was produced at wild-type levels, but cell surface expression was greatly reduced in our cell culture experiments, similar to findings for an Mc1r variant in the lizard *Sceloporus* *undulatus* [33]. Improper processing, failure to maintain conformation, and intracellular retention of the mutant proteins most likely explain the reduced function in light of normal overall expression levels [34,35].

In other species, associations between mutant alleles of *Mc1r* and pigmentation phenotypes (e.g., eumelanic versus not eumelanic) are very strong, including in two other birds with Val85Met mutations [6,7]. In contrast, pigeons with 0, 1, or 2 copies of a Met85 allele can be eumelanic or pheomelanic, and this mutation alone does not appear to be sufficient to drive major pigmentation changes. This mutation could be a modifier of pigmentation traits, and it is possible that the categorical phenotypic classes we used in this study are not sufficient to resolve the relevant subtle pigment differences. Controlled genetic crosses could help to better resolve the association between *Mc1r* and color in pigeons, including dissecting the effects of this locus on specific regions of plumage. For example, genetic mapping helped resolve the effects of *Mc1r* variation across different pelage regions of the rodent *Peromyscus* *polionotus* [36,37].

Strong eumelanic phenotypes are associated with Met85-containing alleles of *Mc1r* in the lesser snow goose and red-footed booby, but the functional consequences of the Val85Met substitution in these species have not been tested experimentally. Why might the Val85Met mutation be associated with increased pheomelanism in the pigeon, but increased eumelanism in other birds? One possibility is that other species-specific amino acid changes in Mc1r of the lesser snow goose and red-footed booby compensate for lower functionality caused by the Val85Met substitution. However, mutations in the same amino acid positions of Mc1r tend to have similar effects on protein function across taxa, irrespective of other mutations in the gene [8]. Another possibility is that Met85 alleles in the lesser snow goose and red-footed booby are not actually responsible for pigmentation variation. Instead, eumelanism might be caused by cis-regulatory variants on the same haplotypes as Met85 substitutions, or by alleles of other genes in linkage disequilibrium with Met85 alleles. In these cases, the Val85Met mutation would serve as a marker for the causative haplotype, rather than be the causative mutation itself. Functional testing of Met85 alleles of the lesser snow goose and red-footed booby orthologs of Mc1r will help distinguish among these possibilities.

*Mc1r* plays a key role in pigment variation in many vertebrate species, so it is intriguing to consider why this locus is a frequent target of selection. Single nucleotide coding mutations in *Mc1r* can have profound effects on pigmentation phenotypes, and such changes are thought to have few deleterious, pleiotropic effects on other cellular and developmental processes [3,38]. However, the role of *Mc1r* is not limited to pigment synthesis, as this gene is also involved in pain perception and immune response, for example 10,39. While it appears likely that *Mc1r*-mediated pigmentation changes are indeed common in vertebrate evolution, another factor in its perceived importance is almost certainly a discovery bias [38,40]. The known involvement of *Mc1r* in color variation in numerous species; the prevalence of easily detectable coding changes; and the ease of sequencing this compact (~1 kb), conserved, single exon gene make *Mc1r* an attractive locus to assay for associations between genotype and color phenotype. That is, more searching has probably led to more discoveries, and studies that do not find associations are probably underreported (but see 41–44 for a review in birds).

Important insights about molecular mechanisms underlying vertebrate diversity have come from studies of domestic animals, and analyses of pigmentation variants have been especially fruitful in both birds and mammals [2,3,45,46]. In pigeons, studies of other candidate genes and forward approaches using genetic crosses and genome-wide association mapping hold promise for dissecting the molecular basis of pigmentation diversity in a classic study system [24,47], and potentially for birds in general.
